# An exceptional Albanian family with seven children presenting with dysmorphic features and mental retardation: maternal phenylketonuria

**DOI:** 10.1186/1471-2431-5-5

**Published:** 2005-04-05

**Authors:** Ina Knerr, Johannes Zschocke, Stefan Schellmoser, Hans G Topf, Corina Weigel, Jörg Dötsch, Wolfgang Rascher

**Affiliations:** 1 Children and Youth Hospital, University of Erlangen-Nuremberg, Loschge Street 15, 91054 Erlangen, Germany; 2 Institute of Human Genetics, University of Heidelberg, Im Neuenheimer Feld 366, 69120 Heidelberg, Germany; 3 Paediatric Practice, Demut Street 21, 9000 St. Gallen, Switzerland

**Keywords:** Maternal phenylketonuria, phenylalanine, phenylketonuria, pregnancy outcome

## Abstract

**Background:**

Phenylketonuria is an inborn error of amino acid metabolism which can cause severe damage to the patient or, in the case of maternal phenylketonuria, to the foetus. The maternal phenylketonuria syndrome is caused by high blood phenylalanine concentrations during pregnancy and presents with serious foetal anomalies, especially congenital heart disease, microcephaly and mental retardation.

**Case presentation:**

We report on an affected Albanian woman and her seven children. The mother is affected by phenylketonuria and is a compound heterozygote for two pathogenetic mutations, L48S and P281L. The diagnosis was only made in the context of her children, all of whom have at least one severe organic malformation. The first child, 17 years old, has a double-chambered right ventricle, vertebral malformations and epilepsy. She is also mentally retarded, microcephalic, exhibits facial dysmorphies and small stature. The second child, a girl 15 years of age, has severe mental retardation with microcephaly, small stature and various dysmorphic features. The next sibling, a boy, died of tetralogy of Fallot at the age of three months. He also had multiple vertebral and rib malformations. The subsequent girl, now eleven years old, has mental retardation, microcephaly and epilepsy along with facial dysmorphy, partial deafness and short stature. The eight-year-old child is slightly mentally retarded and microcephalic. A five-year-old boy was a premature, dystrophic baby and exhibits mental retardation, dysmorphic facial features, brachydactyly and clinodactyly of the fifth finger on both hands. Following a miscarriage, our index case, the youngest child at two years of age, is microcephalic and mentally retarded and shows minor facial anomalies. All children exhibit features of phenylalanine embryopathy caused by maternal phenylketonuria because the mother had not been diagnosed earlier and, therefore, never received any diet.

**Conclusion:**

This is the largest family suffering from maternal phenylketonuria reported in the literature. Maternal phenylketonuria remains a challenge, especially in woman from countries without a neonatal screening program. Therefore, it is mandatory to be alert for the possibility of maternal phenylketonuria syndrome in case of a child with the clinical features described here to prevent foetal damage in subsequent siblings.

## Background

Phenylketonuria (PKU; OMIM *261600) is an autosomal recessive disorder of phe metabolism which can cause severe damage to the patient or, in the case of maternal PKU, to the offspring. The teratogenic effects of elevated maternal phe levels was first recognised in the mid nineteen sixties, at a time when routine newborn screening and diet treatment of PKU was being established in most developed countries [Mabry et al., 1966]. Typical features in offspring with phe embryopathy include microcephaly, mental retardation and heart malformation. The severity of maternal PKU syndrome is proportional to maternal blood phe concentrations, and a strict dietary control prior to conception and throughout pregnancy is mandatory to prevent congenital foetal anomalies [Rouse et al., 2000].

With our case report on an exceptional Albanian family, we wish to highlight the problem of untreated or undiagnosed PKU in adult women, resulting in the risk of severe maternal PKU syndrome in children.

## Case presentation

**Figure 1:**
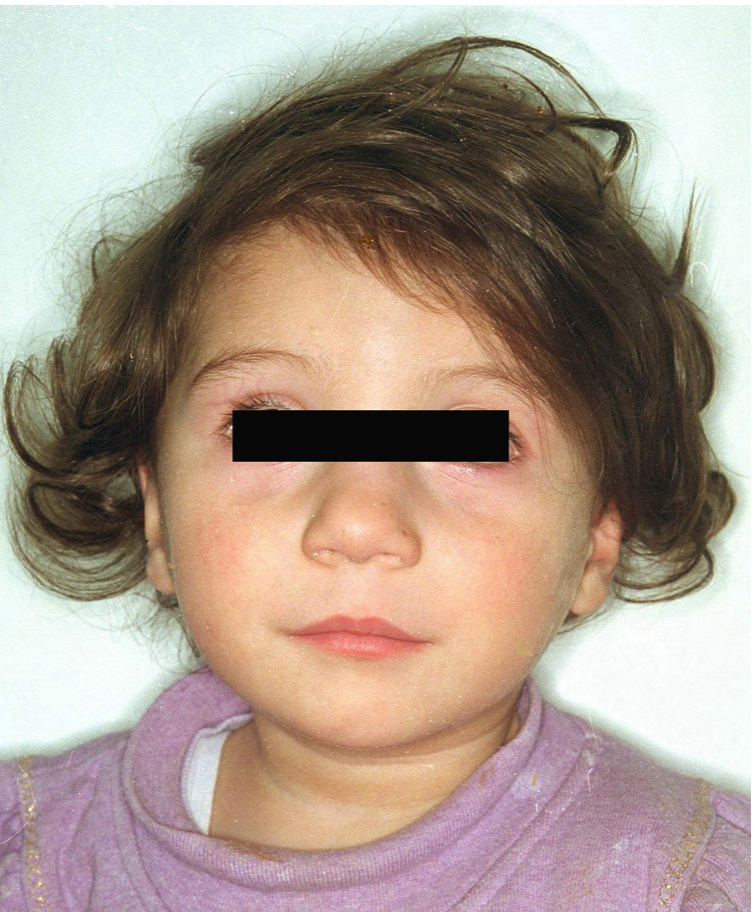
Portrait of the index patient.

Our index case, a 2-year-old girl, is the 7^th ^living child born to her mother (Figure 1). She was admitted to our hospital for further diagnostic work-up. Her birth weight was below 2500 g, as was the case for all her siblings. Additionally, she showed microcephaly, mental retardation and facial anomalies (long underdeveloped philtrum, high palate, anteverted nostrils). She also exhibited a large diastase of the abdominal rectus muscle.

Case 1, the oldest child of the non-consanguine family, a 17-year-old girl, has a double-chambered right ventricle, multiple vertebral malformations of the thoracic and lumbal spine and epileptic seizures. She also shows severe mental retardation, microcephaly, facial dysmorphology (long underdeveloped philtrum, broad nasal bridge, micrognathism, high palate, divergent strabism) and stunted growth.

Case 2, the 15 year-old sister, presented with severe mental retardation, microcephaly, facial dysmorphology (long underdeveloped philtrum, micrognathism, high palate, divergent strabism) and stunted growth. In addition, he has brachydactyly and clinodactyly of the fifth fingers of both hands.

**Figure 2:**
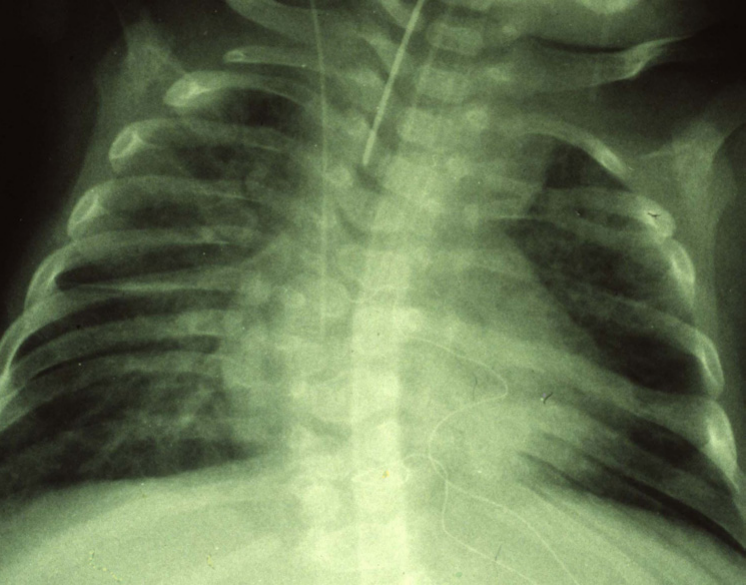
Chest x-ray of case 3 who died of Fallot's tetralogy. The picture exhibits vertebral and rib malformations and fusions along with thoracic scoliosis.

Case 3, a dystrophic boy, died at the age of 3 months of Fallot's tetralogy. He also had vertebral malformations such as hemivertebrae, rib fusions and thoracic scoliosis (Figure 2).

Case 4, an 11-year-old girl, presented with microcephaly and facial dysmorphology (long underdeveloped philtrum, broad nasal bridge, micrognathism, dysplastic ear helices, strabism), partial deafness, mental retardation, absence epilepsy and stunted growth.

Case 5, an 8-year-old girl, was slightly mentally retarded and microcephalic, with minor facial anomalies and short stature.

Case 6, a 5-year-old boy, was a preterm baby and small for gestational age. He also presented with microcephaly, facial dysmorphology and micrognathism. Brachydactyly and clinodactyly of the fifth fingers of both hands were noted.

The mother had one miscarriage between her 6^th ^child and our index patient, case 7. The stillbirth occurred in the 7^th ^month of pregnancy.

Further diagnostic work-up in case 1 to 7 was unremarkable. Because of the characteristic features of all her children, maternal serum phe concentration was analysed. The blood phe level was greatly elevated with 1560 μmol/L (normal < 90 μmol/L). Urinary organic acid analysis showed high excretion of phenylpyruvic, phenylacetic, hydroxyphenylacetic and phenyllactic acids as well as mandelic acid. Mutation analysis revealed that the mother was compound hetereozygous for the mutations L48S and P281L in the PAH gene. This disease was previously unknown to the mother. A newborn screening program had not yet been established in 1963 in Albania when the mother was born. The mother did not wish to maintain a phe-restrictive or protein-restrictive diet, and the children were unavailable to us for a long-term follow-up.

## Conclusion

The case histories of the patients reported here provide further information on the maternal PKU syndrome, which causes heart malformation, microcephaly, mental retardation and stunted growth, respectively [Koch et al., 2003;Lee et al., 2003;Levy et al., 2001;Rouse et al., 1997], since it is the largest family suffering from maternal PKU reported in the literature. There are several reports of undiagnosed maternal PKU in the English language literature [Starostecka et al., 2001;Hanley et al., 1999], however, none with as many as 7 offspring. The disease was previously unknown to the mother who had visited a primary school and was not noted to have subnormal intelligence. The mother did not speak German but was socially integrated. Interestingly, the couple reported restricted food, especially of protein-rich meals, in Albania due to their low social status. However, seven of the children were born in Germany. All offspring had normal phe serum concentrations. The genotype identified in the mother of our patients, compound heterozygosity for the common mutations L48S and P281L, is associated with complete loss of enzyme activity of PAH and a classical PKU phenotype [Zschocke, 2003]. Since untreated PKU does not, in all patients, lead to severe neurological symptoms and profound mental handicap, as in the mother described here, there is a continuing need to be alert for the possibility of maternal PKU syndrome to prevent phe embryopathy in subsequent siblings. Although the condition is well known, it remains a problem, particularly in women born in countries without neonatal screening programs. However, the incidence of undiagnosed maternal PKU is currently estimated at 1 case per 100,000 births or even higher in Europe and the US [Hanley et al., 1999].

In conclusion, maternal blood phe concentrations should be investigated in the case of an otherwise unexplained neonatal syndrome presenting with microcephaly, facial dysmorphy and heart malformation, particularly in mothers born in countries without neonatal screening programs even if there is no obvious maternal mental retardation. Our case report clearly demonstrates that maternal PKU is still a challenge to be faced.

## Competing interests

The author(s) declare that they have no competing interests.

## Authors' contributions

Knerr, principal investigator; Zschocke, molecular studies, laboratory investigations; Schellmoser, clinical studies; Topf, Weigel, manuscript discussion; Dötsch, Rascher, scientific discussion.

## Pre-publication history

The pre-publication history for this paper can be accessed here:


